# Phosphodiesterase Type 5 Inhibitor Sildenafil Decreases the Proinflammatory Chemokine CXCL10 in Human Cardiomyocytes and in Subjects with Diabetic Cardiomyopathy

**DOI:** 10.1007/s10753-016-0359-6

**Published:** 2016-05-10

**Authors:** Luigi Di Luigi, Clarissa Corinaldesi, Marta Colletti, Sabino Scolletta, Cristina Antinozzi, Gabriella B. Vannelli, Elisa Giannetta, Daniele Gianfrilli, Andrea M. Isidori, Silvia Migliaccio, Noemi Poerio, Maurizio Fraziano, Andrea Lenzi, Clara Crescioli

**Affiliations:** Department of Movement, Human and Health Sciences, University of Rome “Foro Italico”, Rome, Italy; Department of Medical Biotechnologies, Anesthesia and Intensive Care, University of Siena, Siena, Italy; Department of Experimental and Clinical Medicine, University of Florence, Florence, Italy; Department of Experimental Medicine, Sapienza University of Rome, Rome, Italy; Department of Biology, University of Rome “Tor Vergata”, Rome, Italy

**Keywords:** inflammation, T helper 1, cardiomyopathy, CXCL10, PDE5 inhibition

## Abstract

T helper 1 (Th1) type cytokines and chemokines are bioactive mediators in inflammation underling several diseases and co-morbid conditions, such as cardiovascular and metabolic disorders. Th1 chemokine CXCL10 participates in heart damage initiation/progression; cardioprotection has been recently associated with sildenafil, a type 5 phosphodiesterase inhibitor. We aimed to evaluate the effect of sildenafil on CXCL10 in inflammatory conditions associated with diabetic cardiomyopathy. We analyzed: CXCL10 gene and protein in human cardiac, endothelial, and immune cells challenged by pro-inflammatory stimuli with and without sildenafil; serum CXCL10 in diabetic subjects at cardiomyopathy onset, before and after 3 months of treatment with sildenafil vs. placebo. Sildenafil significantly decreased CXCL10 protein secretion (IC_50_ = 2.6 × 10^−7^) and gene expression in human cardiomyocytes and significantly decreased circulating CXCL10 in subjects with chemokine basal level ≥ 930 pg/ml, the cut-off value as assessed by ROC analysis. In conclusion, sildenafil could be a pharmacologic tool to control CXCL10-associated inflammation in diabetic cardiomyopathy.

**Electronic supplementary material:**

The online version of this article (doi:10.1007/s10753-016-0359-6) contains supplementary material, which is available to authorized users.

## INTRODUCTION

Nowadays, there is growing evidence on a tight association between cardiac dysfunction and T helper (Th)1-driven inflammation [1–3], with the specific involvement of some cytokines and chemokines, such as tumor necrosis factor (TNF)α, monocyte chemoattractant protein (MCP)-1 or CCL2, macrophage inflammatory protein (MIP)1-β or CCL4, interferon (IFN)-γ-inducible protein-10, IP-10 or CXCL10, interleukin (IL)-8 or CXCL8, IL-1β, IL-6, IL-12, IL-23 [2, 4–11].

In particular, the chemokine CXCL10 has been shown to play a pivotal role in early Th1-driven processes underlying heart damage initiation and progression, *i.e*., during cardiac transplantation, myocarditis, and cardiopulmonary bypass [12–17]. The level of pretransplant serum CXCL10 is a predictive biomarker of cardiac damage and acute organ rejection in heart transplant recipients [12]; furthermore, CXCL10 can be induced and released by human cardiac cells under inflammatory challenge and likely contributes to establish and maintain a self-inflammatory loop between blood and local tissues [12–16]. This small chemokine is, indeed, one of the major trigger of activation and polarization of Th1 inflammatory response both at systemic and tissue level [12, 13, 18–20].

It is quite a recent acquisition that some Th1-driven processes can be counteracted by the inhibitors of phosphodiesterase type 5 (PDE5i) [21]. This class of drugs, which includes sildenafil, vardenafil, tadalafil, and avanafil, is widely used as on-demand treatment for erectile dysfunction (ED) because of the significant vasodilatory effect achieved through PDE5 inhibition and cyclic guanosine monophosphate (cGMP) stabilization in vascular beds [22–27]. In view of those vasoactive effects, PDE5 inhibition has been hypothesized as a therapeutic approach for a number of cardiovascular disturbances as well. Sildenafil, in particular, has been shown to exert cardioprotective effects after ischemia/reperfusion injuries, reduce infarct size or ventricular arrhythmias [24, 28, 29], ameliorate heart failure (HF) patients with reduced ejection fraction (EF), and improve cardiac functional capacity and geometry in subjects with diabetic cardiomyopathy [30, 31]. Interestingly, studies on effectiveness show that sildenafil retains better cardiovascular profile vs. the other PDE5i [32]. Thus, sildenafil seems a good candidate to be a therapeutic tool in cardiovascular medicine [25].

It is well-known that vascular bed is the “standard” target of PDE5i [33, 34].

The purpose of this study is to verify whether sildenafil could target CXCL10 in human cardiomyocytes challenged by Th1-type-inflammatory stimuli; IL-6 and IL-8, both involved in inflammation and cell damage in cardiac disease [35, 36], have been also investigated. Methylprednisolone (MeP), cyclosporine (CsA), and mycophenolate (MPA) were used for comparison. We also analyzed the effect of sildenafil onto CXCL10 in human endothelial and different subtypes of immune cells (human PBMC, monocytes, macrophages) in the same experimental conditions. Furthermore, we aimed to investigate circulating level of CXCL10 in subjects with diabetic cardiomyopathy, a Th1-driven comorbid condition, before and after 3 months sildenafil intake vs. placebo.

## MATERIALS AND METHODS

### Chemicals

Dulbecco modified eagle medium (DMEM)/Ham’s F-12 medium (1:1) with and without phenol red, RPMI 1640, phosphate buffered saline Ca^2+^/Mg^2+^-free (PBS), bovine serum albumin (BSA) fraction V, antibiotics, NaOH, EDTA-trypsin solution, Bradford reagent, methylprednisolone (MeP), cyclosporine A (CsA) and mycophenolic acid (MPA), phytohemagglutinin (PHA), PDE5 inhibitor sildenafil citrate salt, PDE4 inhibitor rolipram, PDE3 inhibitor cilostamide, PDE2 inhibitor erythro-9-(2-hydroxy-3-nonyl)adenine (EHNA), lipopolysaccharides (LPS) from *Escherichia coli* 0111:B4 were from Sigma-Aldrich Corp. (St. Louis, MO, USA). EGM™ Endothelial Growth Medium & EGM™ BulletKit™ were from Lonza; fetal bovine serum (FBS) and fetal calf serum (FCS) were from Hyclone (Logan, UT, USA). Recombinant Human IFN-γ and recombinant Human TNF-α were from Peprotech® (RockyHill, NJ, USA). L-glutamine was from Gibco Laboratories (Grand Island, NY). Granulocyte-macrophage colony-stimulating factor (GM)-CSF, ELISA kit for CXCL10, IL-8, and IL-6 measurement were from R&D Systems (Minneapolis, MN, USA). For RNA extraction, TRIzol RNA isolation reagent was purchased by Ambion™; for reverse transcription 10 mM dNTP mix, random primers, RNaseOUT™ Ribonuclease inhibitor and SuperScript® III Reverse were purchased from Invitrogen. SYBR® Green PCR Master Mix for qPCR was from Life Technologies™ (Applied Biosystems®). Ficoll-Paque PLUS was from Amersham Bioscences (Little Chalfont, UK). Trypan blue 0.5 % was from Euroclone® (UK). Plastic ware for cell cultures and disposable filtration units for growth media preparation were purchase from Corning (Milan, Italy). Polyclonal rabbit anti-PDE5 and monoclonal mouse anti-β-actin were from Santa Cruz (CA, USA). All reagents for SDS-PAGE were from Millipore (Billerica, MA, USA). AutoMACS® Rinsing Solution, MACS® BSA Stock Solution, MicroBeads conjugated to monoclonal anti-human CD14 antibodies (isotype: mouse IgG2a), LS MACS® columns, and MidiMACS® separator were from Miltenyi Biotec (Bergisch Gladbach, Germany). Fluorochrome-conjugated CD14 antibody for flow cytometric analysis (CD14-FITC), Fluorescence-activated cell sorting (FACS) Calibur and CellQuest software for data analysis were from Becton Dickinson (Vienna, Austria).

### Cell Cultures

Human fetal cardiomyocytes (Hfcm) and human fetal aortic endothelial cells (Hfaec) were obtained from cardiac tissues or aortic ascendant tracts collected after voluntary abortion (10–12 weeks of gestation) characterized and maintained as described elsewhere [15, 20]. Legal abortions were performed in authorized hospitals, and certificates of consent were obtained. The use of human fetal tissue for research purposes conforms with the principles outlined in the Declaration of Helsinki and was approved by the committee for investigation in humans of the Azienda Ospedaliero-Universitaria Careggi, Florence, Italy (protocol no. 6783–04).

Peripheral blood mononuclear cells (PBMC) were isolated from buffy coats obtained from healthy adult anonymous donors in accordance with local ethical committee; approval by Azienda Policlinico Umberto I Rome Italy, accordance with the principles outlines in the Declaration of Helsinki and written consents were obtained. Briefly, heparinized blood, collected from peripheral vein, was centrifuged on Ficoll-Hystopaque gradient following manufacture’s protocol. PBMC were cultured in RPMI supplemented with 10 % FBS, 2 mmol/l L-glutamine and antibiotics.

Monocytes were purified from total PBMC by negative selection using antibodies conjugated to magnetic beads and cultured in RPMI supplemented with 10 % FBS, 2 mmol/l L-glutamine and antibiotics. After isolation, 2 × 10^6^ cells were washed twice with PBS and stained with FITC-conjugated anti-CD14 for 30 min. After two washing steps, cells were analyzed on a flow cytometer. Cell population purity (85–90 %) was assessed by sorting the CD14-positive (CD14^+^) cells. Macrophages were obtained from highly purified CD14^+^ peripheral blood monocytes seeded onto 96-well round bottom plates in their growth medium and treated for 5 days with (GM)-CSF (35 ng/ml). Cell cultures in their specific growth medium were maintained in a fully humidified atmosphere of 95 % air and 5 % CO_2_.

### Subjects

Frozen samples from 30 subjects with diabetic cardiomyopathy (DCM) enrolled and described in a previous study were analyzed (Clinical Trial Registration—URL: http://www.clinicaltrials.gov. Unique identifier: NCT00692237) [31]. The protocol was approved by Hospital Ethics Committee Policlinico Umberto I—Sapienza University Hospital of Rome, and written informed consent was obtained. This was a randomized controlled trial with patients allocated to receive 100 mg/day sildenafil for 3 months or placebo [31]. A brief description of the protocol is here reported. Eligible men with type 2 diabetes were recruited from the outpatient of Policlinico Umberto I—Sapienza University Hospital of Rome, the inclusion criteria were: age 35–75 years; T2DM > 1 year; glycated hemoglobin (HbA1c) < 10 %; normal blood pressure (BP) or treated hypertension with achievement of a target of ≤130/80 mmHg; BMI < 40. The exclusion criteria were: use of exogenous insulin, thiazolidinediones, or spironolactone; prior or current use of PDE5i; substance abuse; history of cardiovascular disease, proliferative retinopathy, autonomic neuropathy; symptoms or signs of ischemic heart disease during cardiac evaluations at enrollment; contraindications to sildenafil use or CMR imaging. Concomitant medications (anti-hypertensives, statins, oral anti-diabetics, *etc*.) were not changed between the months prior to the study and 1 month after its completion. All blood samples were collected from peripheral vein and serum was obtained by centrifugation (3,000 rpm for 10 min at 4 °C); aliquots were stored at −80 °C until analyzed. Analysis was performed on the active arm of the trial (sildenafil treatment).

Two additional groups of subjects, matched for sex and age, were analyzed for comparisons: eight subjects affected by diabetes without signs of cardiac impairment (DM) and eight subjects without diabetes, but with cardiac hypertrophy (IC). Characteristics of baseline the study population are reported in Table 1. Written informed consent was collected for all subjects.

**Table 1:** Metabolic and Clinical Characteristics of the Study Populations

Variable	Diabetic cardiomyopathy sildenafil arm^a^	Diabetic cardiomyopathy placebo arm	Diabetes mellitus without cardiomyopathy	Non diabetic hypertensive cardiomyopathy
Numbers	30	16	8	8
Age (years)	61.26 ± 1.42	60.72 ± 1.33	62.65 ± 4.29	63.40 ± 4.10
BMI (kg/m^2^)	28.41 ± 0.89	27.60 ± 0.85	27.38 ± 1.23	25.77 ± 1.31*
Glycemia (mmol/L)	8.38 ± 1.96	8.18 ± 1.56	7.98 ± 1.65	5.08 ± 0.65*
HOMA-index	6.31 ± 0.78	7.55 ± 0.84	7.98 ± 0.52	5.08 ± 0.21*
HbAlc (%)	7.95 ± 0.23	7.33 ± 0.27	6.90 ± 0.49	5.85 ± 0.11*
Mean systolic BP (mmHg)	136.11 ± 2.21	131.45 ± 2.85	129.14 ± 3.64	138.87 ± 3.96
Mean diastolic BP (mmHg)	79.66 ± 1.60	78.94 ± 1.43	77.68 ± 1.40	81.75 ± 2.32
LVMi (g/m^2^)	124.60 ± 4.75	114.15 ± 5.96	88.62 ± 7.69†	110.68 ± 8.55
EDVi (mL/m^2^)	61.36 ± 1.74	60.76 ± 2.29	64.26 ± 4.11	59.16 ± 4.15
Ejection fraction (%)	61.59 ± 1.52	59.11 ± 1.90	66.80 ± 1.81	63.12 ± 2.75
NT proBNP (pg/ml)	70.00 ± 11.88	72.23 ± 18.52	66.38 ± 4.69	81.73 ± 10.90

Values are expressed as mean ± SEM

^a^All subjects (responders and no-responders)

*P < 0.05 (IC vs. DM), †*P* < 0.05 (DCM vs. DM)

### Cytokine Secretion Assay

For CXCL10 secretion assay in Hfcm and Hfaec and for IL-8 and IL-6 assay in Hfcm, 4,000 cells/well were seeded onto 96-well flat bottom plates and maintained for 24 h in DMEM/Ham’s F-12 medium (1:1) 10 % FBS or Endothelial Growth Medium (EGM™), respectively. After overnight starvation (medium without serum and without phenol red), cells were stimulated for 24 h with IFNγ (1,000 U/ml) + TNFα (10 ng/ml) with or without sildenafil (1 μM), MeP (250 ng/ml), CsA (250 ng/ml), or MPA (26 μg/ml) in serum free medium with 0.1 % BSA. Cells in serum-free medium containing 0.1 % BSA and vehicle were used as control. PBMC, 200,000 cells/well, were seeded onto 96-well round bottom plates in their growth medium and stimulated for 48 h with 2 % PHA with or without sildenafil (1 μM), MeP (250 ng/ml), CsA (250 ng/ml), or MPA (26 μg/ml) in RPMI supplemented with 10 % FBS, 2 mmol/l L-glutamine and antibiotics. Monocytes and macrophages, 200,000 cells/well, were seeded onto 96-well round bottom plates in their growth medium and stimulated for 48 h with LPS (200 ng/ml) with or without sildenafil (1 μM), MeP (250 ng/ml), CsA (250 ng/ml), or MPA (26 μg/ml) in RPMI supplemented with 10 % FBS, 2 mmol/l L-glutamine and antibiotics.

Cells in growth medium with vehicle were used as control. The drug concentrations were selected based on their near-therapeutic doses, according to their pharmacokinetics (Cmax and area under the time concentration curves, AUC).

For dose–response assays, Hfcm, after overnight starvation, were incubated for 24 h with IFNγ (1,000 U/ml)+TNFα (10 ng/ml) with or without sildenafil (1 × 10^−7^, 2.5 × 10^−7^, 5 × 10^−7^, 1 × 10^−6^, 2.5 × 10^−6^, 5 × 10^−6^, 1 × 10^−5^, 2,5 × 10^−5^ M) or MPA (8.1 × 10^−7^, 4.1 × 10^− 6^, 8.1 × 10^− 6^, 2.05 × 10^− 5^, 4.1 × 10^−5^, 8.1 × 10^−5^, 1.6 × 10^−4^, 3.2 × 10^−4^ M).

For assays with different specific inhibitors of PDE subtypes, Hfcm were seeded, maintained, and starved in the same conditions as described above. Cells were treated for 24 h with IFNγ (1000 U/ml)+TNFα (10 ng/ml) with or without sildenafil (1 μM), rolipram (10 μM), cilostamide (1 μM), EHNA (10 μM), in serum free medium with 0.1 % BSA. Cells in serum-free medium containing 0.1 % BSA and vehicle were used as control.

Supernatants from each experimental setting were harvested, centrifuged, and stored to −20 °C until performing ELISA. All experiments were performed in triplicate with at least four different cells preparations.

### ELISA Assays

CXCL10, IL-8, and IL-6 levels were measured in cell culture supernatants using commercially available kits, according to the manufacturer’s recommendations.

The sensitivity ranged from 0.41 to 4.46 pg/ml for CXCL10, less than 0.70 pg/ml for IL-6, from 1.5 to 7.5 pg/ml for IL-8. The intra- and inter-assay coefficients of variation were: 3.1 and 6.7 % for CXCL10, 4.4 and 3.7 % for IL-6, 4.6 and 8.1 % for IL-8. Quality control pools of low, normal, and high concentrations for all parameters were included in each assay. Protein measurement to normalize the amount of secreted cytokines was performed as reported elsewhere [19]. Data were expressed as percent of inhibition, calculated on IFNγ + TNFα-, PHA- or LPS-induced release, taken as 100 %.

### Western Blot Analysis

For protein analysis, Hfcm and Hfaec were seeded and maintained in the same conditions as previously reported [15, 20]. For PBMC and monocytes/macrophages protein analysis, 3 × 10^6^ cells were seeded onto 100-mm dishes or 25-cm^2^ cell cultures flask, respectively, and maintained in RPMI 1640 medium. Protein concentration measurement was performed with Bradford Reagent. Protein aliquots (20 μg) were processed, loaded onto 10 % SDS-PAGE, transferred on nitrocellulose membranes, and incubated with primary Abs appropriately diluted in Tween Tris-buffered saline (TTBS; for anti-PDE5 and anti-β-actin 1:1,000), followed by peroxidase-conjugated secondary IgG (1:10,000). Proteins were revealed by the enhanced chemiluminescence system (ECL plus; Millipore). Image acquisition was performed with Image Quant Las 4000 software (GE Healthcare). Western blot analysis was performed for three/four independent experiments with different cell preparations.

### RNA Extraction, Reverse Transcription and Real-Time Quantitative PCR

For mRNA analysis: 35,000 Hfcm or Hfaec were seeded in 35-mm culture dishes and maintained for 24 h in their growth medium; after 12-h starvation (medium without serum and without phenol red), cells were stimulated for 24 h with a combination of IFNγ (1,000 U/ml) + TNFα (10 ng/ml) with or without sildenafil (1 μM), MeP (250 ng/ml), CsA (250 ng/ml), or MPA (26 μg/ml) in serum-free medium with 0.1 % BSA, cells in serum-free medium containing 0.1 % BSA and vehicle were used as control; 200,000 PBMC were seeded onto 96-well round bottom plates in their growth medium and stimulated for 48 h with 2 % PHA with or without sildenafil (1 μM), MeP (250 ng/ml), CsA (250 ng/ml), or MPA (26 μg/ml), cells in growth medium and vehicle were used s control. Total RNA was extracted from cultured cells using TRIzol® RNA Isolation Reagents (Ambion™) according to the manufacturer’s instructions. Single-stranded cDNA was obtained by reverse transcription of 1 μg of total RNA. RT-qPCRs were performed using 7500 Real Time System (Applied Biosystems®) with SYBR-green fluorophore; 40 ng of cDNA were used as template and cycling parameters were 95 °C for 10 min, followed by 40 cycles of 15 s at 95 °C, 1 min at 60 °C, 30 s at 95 °C, 15 s at 60 °C. Fluorescence intensities were analyzed using the manufacturer’s software (7500 Software v2.05) and relative amounts were obtained using the 2^−∆∆Ct^ method and normalized for the ß-actin. Data are expressed as fold increase vs. IFNγ + TNFα- or PHA-induced expression taken as 1. Primers for CXCL10 were: forward (TTCCTGCAAGCCAATTTTGT) and reverse (ATGGCCTTCGATTCTGGATT); for β-actin, forward (CTGAACCCCAAGGCCAAC) and reverse (AGCCTGGATAGCAACGTACA).

### Cell Viability

For cell viability assays: Hfcm and Hfaec (4,000 cells/well) were seeded in 96-well plates, maintained in phenol red- and serum-free medium overnight and incubated in serum-free medium containing 0.1 % BSA with sildenafil (1 μM) for 24–48 h; cells in serum-free medium containing 0.1 % BSA and vehicle were used as control.

PBMC (150,000 cells/well) were seeded in 96-well plates in their growth medium and stimulated with PHA (2 %) with or without sildenafil (1 μM) for 24–48 h. Control cells were in growth medium with vehicle and PHA. In each cell type, sildenafil was added each day. Cell viability was assessed each day by trypan blue (0.05 % *v*/*v* solution in PBS) exclusion, mixed in a ratio of 1:1. Cell number counting was assayed by hemocytometer; only cells that excluded trypan blue dye were included in the analysis. The number of viable cells at each time point was derived averaging at least five different fields for each well and each experimental point was repeated in duplicate or triplicate. Results are expressed as percent of cells at time zero (time of seeding). Experiments were performed four times with different cell preparations.

### Serum CXCL10 Assay

Serum levels of CXCL10 were measured using a magnetic bead-based multiplex assay (Bio-Plex Pro™ Human Cytokine, Chemokine and Growth factor assay, Bio-Rad laboratories, Inc.) according to the manufacturer’s protocol. A broad sensitivity range of standards (between 1.95 and 95,000 pg/ml; Bio-Rad Laboratories, Inc.) was used to enable the quantization of a dynamic wide range of cytokine concentrations and provide the greatest sensitivity. Data acquisition was performed by Bio-Plex 200 System™ (Bio-Rad Laboratories, Inc.) which uses Luminex fluorescent-bead-based technology (Luminex) with a flow-based dual laser detector with real-time digital signal processing to facilitate the analysis of up to 100 different families of color-coded polystyrene beads and allow multiple measurements of the sample ensuing in the effective quantification of cytokines. Data analysis was performed by Bio-Plex Manager™ 6.0 software (Bio-Rad Laboratories, Inc.). Serum samples were run in triplicate at least twice.

### Statistical Analysis

The statistical analysis was performed using 11.5 SPSS (SPSS Inc, Chicago, IL, USA). The Kolmogorov–Smirnov test was used to test for normal distribution of the data. Continuous data were compared using unpaired Student’s *t* test or Mann–Whitney test when appropriate. Binomial data were compared using chi-square analysis and Fisher’s exact test when appropriate. Receiver operating characteristic (ROC) curve was constructed to identify the best predictive CXCL10 threshold (cut-off value) capable of discriminating between responders and non-responders to sildenafil administration. Briefly, ROC curve gives a graphic representation of the relationship between true-positive fraction (sensitivity, Se) and false-positive fraction (1-specificity, Sp). ROC curve can be assessed by plotting the values of 1-Sp against Se in a squared box, where the ROC’s area under the curve (AUC) is used to measure the performance of a diagnostic test. The AUC lies in the interval 0.5 to 1.0, so that the greater the area, the better the performance of the variable being examined. A *P* value less than 0.05 was considered significant.

Sigmoid curves were performed using GraphPad Prism 5 software (GraphPad Software, Inc., La Jolla, CA, USA) and SPSS 12.0 software package (SPSS for Windows 12.0, SPSS Inc.,Chicago, IL, USA). The Kolmogorov–Smirnov test for normal distribution of the data, one-way analysis of variance (ANOVA), *t* test were applied. A *P* value less than 0.05 was considered significant and corrected for comparison using the Dunnett’s or Bonferroni’s *post hoc* test, where appropriate.

Data were expressed as the mean ± SE.

## RESULTS

### Effect of Sildenafil onto CXCL10 Secretion and Gene Expression in Hfcm, Hfaec, and PBMC After Inflammatory Activation

To verify whether PDE5 inhibition might affect Th1-driven inflammatory response at cellular level, the effect of sildenafil onto CXCL10 release has been evaluated in human cardiac, endothelial, and immune cells challenged by maximal inflammatory stimuli in comparison to MeP, CsA, and MPA; drug concentrations have been selected on the basis of the near therapy doses, according to their pharmacokinetics (Cmax and area under the time-concentration curve, AUC).

**Fig. 1:**
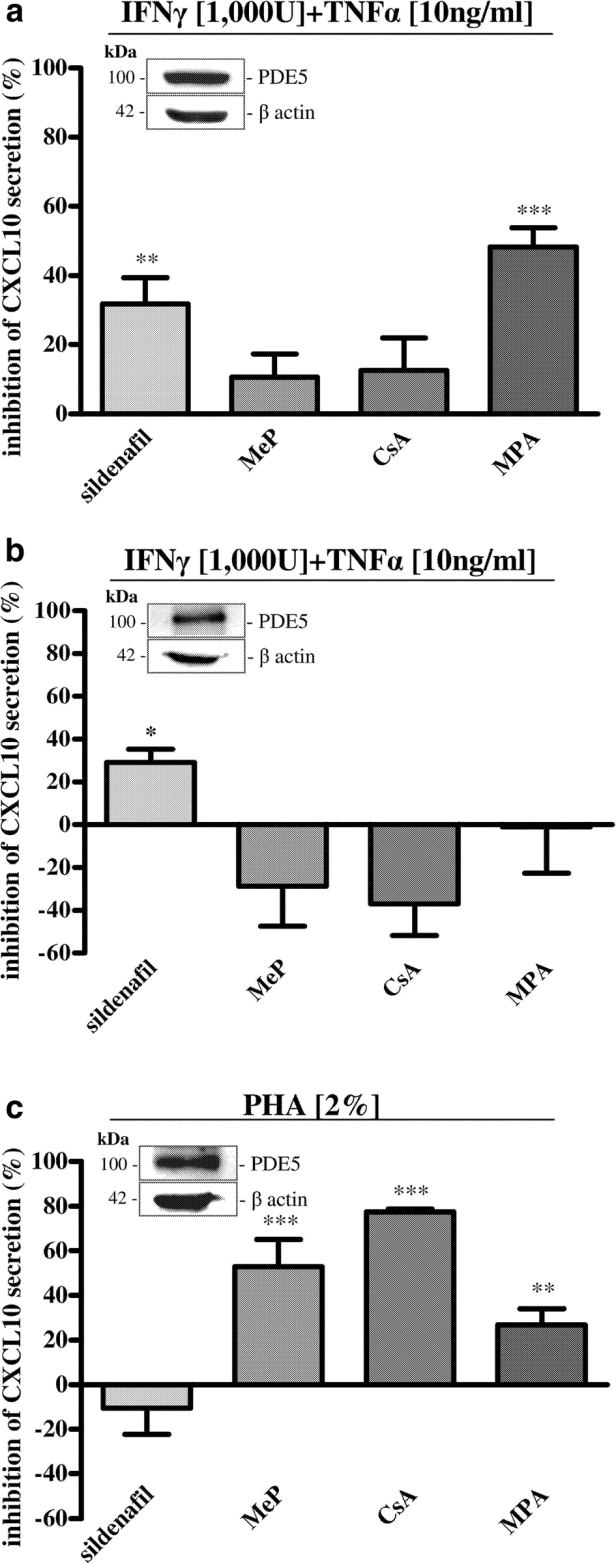
Effect of sildenafil on cytokine-induced CXCL10 secretion in human cells in comparison with different immunosuppressors. **a** In Hfcm, a significant inhibition of IFNγ + TNFα-induced CXCL10 protein secretion has been observed after 24-h incubation with sildenafil or MPA (31.8 ± 7.6 and 48.3 ± 5.5 % inhibition, respectively; **P < 0.01;***P < 0.001 ); no effect has been observed in presence of MeP or CsA. **b** Cytokine-induced CXCL10 protein secretion by Hfaec has been significantly reduced by sildenafil after 24 h (*P < 0.05; 29.1 ± 6.3 % inhibition) while no effect has been achieved in presence of the other drugs. **c** Sildenafil did not affect CXCL10 release by PHA-activated PBMC after 48 h, whereas MeP, CsA and MPA exerted a significant inhibition (MeP: 52.8 ± 12.4 %, CsA: 77.5 ± 1.2 %, MPA: 26.80 ± 7.2 % inhibition; ***P < 0.001 and **P < 0.01). PDE5 protein expression has been detected by western blot in all three cell types investigated (*inset* of **a**, **b**, and **c**). Results (mean ± SE) are expressed as inhibition of CXCL10 secretion percent of IFNγ + TNFα- or PHA-induced release, taken as 100 %. Data are obtained from four to nine experiments using different cell preparations.

In Hfcm (Fig. 1a), sildenafil and MPA significantly inhibited IFNγ + TNFα-induced CXCL10 protein secretion (*P* < 0.01, *P* < 0.001); CsA and MeP did not exert any significant effect. Hfcm express PDE5, as shown by western blot analysis (inset of Fig. 1a).

Sildenafil and MeP significantly inhibited IFNγ + TNFα-induced IL-6 protein secretion (*P* < 0.001), differently from CsA and MPA (Fig. S1 A). Sildenafil did not significantly affect IL-8 protein secretion similarly to MPA, at variance with MeP and CsA (*P* < 0.001 and *P* < 0.05, respectively, Fig. S1 B).

In Hfaec (Fig. 1b), sildenafil significantly reduced IFNγ + TNFα-induced CXCL10 secretion—although to a less extent as compared to Hfcm (*P* < 0.05)—whereas no effect has been achieved with the other immunosuppressants. PDE5 protein expression has been detected in Hfaec (inset of Fig. 1b).

In PBMC, sildenafil did not change PHA-induced CXCL10 release, at variance with the other immunosuppressants (*P* < 0.01 and *P* < 0.001, Fig. 1c). PBMC express PDE5 protein (inset of Fig. 1c).

In monocytes sildenafil, CsA and MPA did not exert any significant effect on LPS-induced CXCL10 release, while MeP induced a significant inhibition (*P* < 0.05, Fig. S2a). None of the tested drugs affected LPS-induced CXCL10 secretion by macrophages (Fig. S2b).

Monocytes purity was assessed by FACS analysis before and after CD14 magnetic beads separation and was around 10 and 90 % of total cell population, respectively (Fig. S2c, left and right representative panels).

mRNA expression specific for CXCL10 after the different drug treatments has been investigated in each tested cell type and reported in Table 2 and related legend. In particular, sildenafil and MPA significantly inhibited CXCL10 mRNA expression in Hfcm (*P* < 0.01 and *P* < 0.05, respectively); no significant effect has been observed in Hfaec and in PBMC after sildenafil.

**Table 2:** CXCL10 Gene Expression in Human Cardiac, Endothelial, and Immune Cells

	mRNA expression (fold increase)			
	Sildenafil	MeP	CsA	MPA
Hfcm	0.80 ± 0.05**	1.15 ± 0.72	1.07 ± 0.15	0.75 ± 0.14*
Hfaec	1.09 ± 0.35	1.23 ± 0.79	0.73 ± 0.1*	0.62 ± 0.07*
PBMC	1.48 ± 0.37	2.80 ± 0.65*	0.15 ± 0.06***	1.72 ± 0.41**

mRNA expression specific for CXCL10 in Hfcm, Hfaec and PBMC is depicted after different drug treatments. In Hfcm, sildenafil and MPA significantly inhibited CXCL10 mRNA expression (0.80 ± 0.05 **P < 0.01 and 0.75 ± 0.14 *P < 0.05 respectively)

In Hfaec, sildenafil did not exert any effect on CXCL10 gene expression; CsA and MPA significantly affected CXCL10 gene expression (0.73 ± 0.1*P < 0.05 and 0.62 ± 0.07 *P < 0.05 respectively)

In PBMC, sildenafil did not modify CXCL10 gene expression at variance with MeP, CsA, and MPA (2.80 ± 0.65 *P < 0.05, 0.15 ± 0.06 ***P < 0.001, 1.72 ± 0.41 **P < 0.01 respectively)

Results derive from at least four different cell preparations and are expressed as fold increase vs. IFNγ + TNFα- or PHA-induced expression, taken as 1

### IC_50_ Determination in Hfcm

**Fig. 2:**
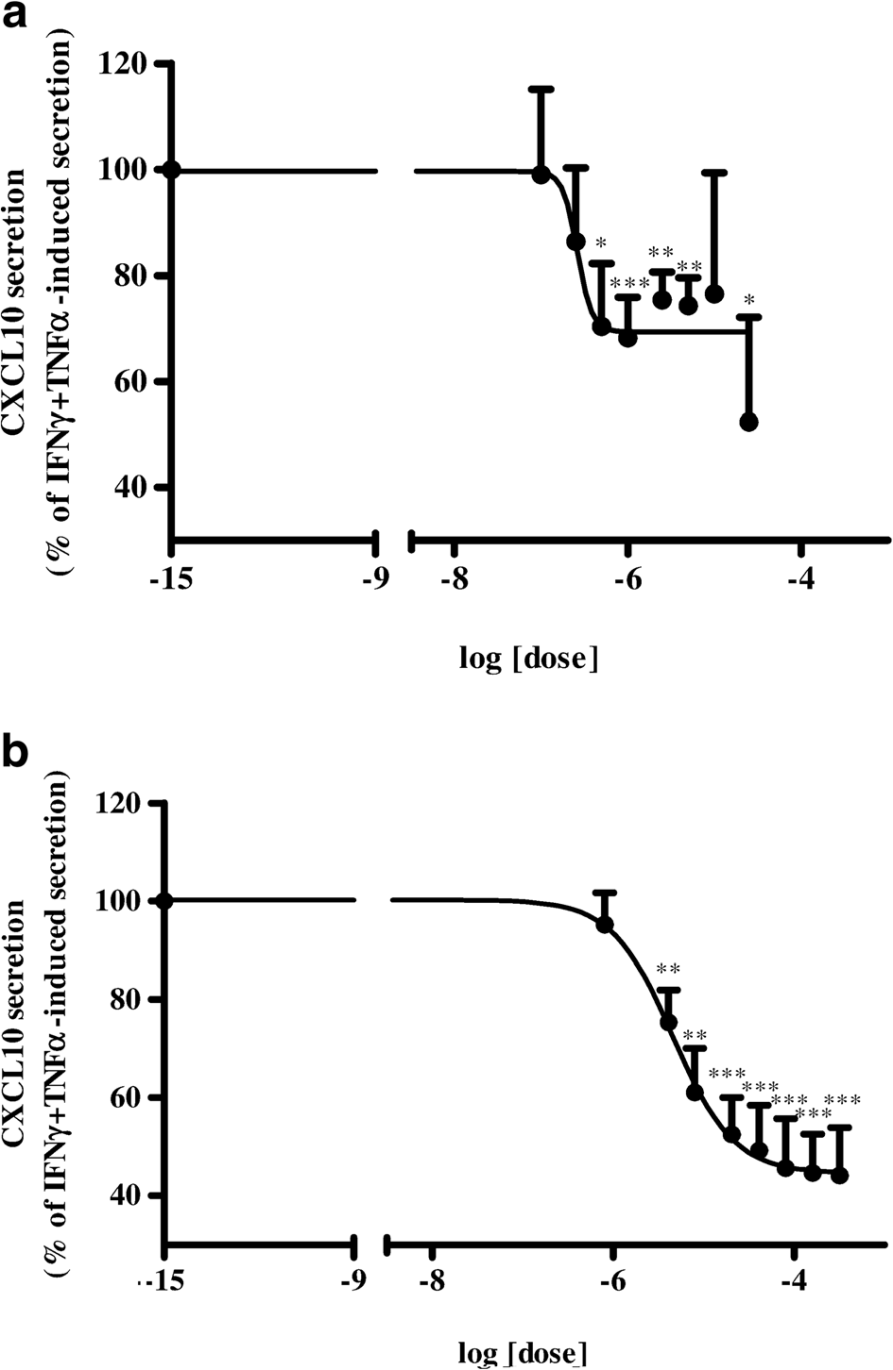
Sildenafil-induced dose-dependent inhibition of CXCL10 secretion and IC_50_ calculation in human cardiomyocytes. **a** Incubation with increasing doses of sildenafil (1 × 10^−7^, 2.5 × 10^−7^, 5 × 10^−7^, 1 × 10^−6^, 2.5 × 10^−6^, 5 × 10^−6^, 1 × 10^−5^, 2.5 × 10^−5^ M) significantly and dose-dependently reduced CXCL10 protein release, starting from 5 × 10^−7^ M, by Hfcm stimulated with IFNγ (1,000 U/ml)+TNFα (10 ng/ml). The IC_50_ was 2.6 × 10^−7^ M. **b** MPA (8.1 × 10^−7^, 4.1 × 10^−6^, 8.1 × 10^−6^, 2.05 × 10^−5^, 4.1 × 10^−5^, 8.1 × 10^−5^, 1.6 × 10^−4^, 3.2 × 10^−4^ M), used for comparison, has been confirmed to induce a dose-dependent inhibition of cytokine-induced CXCL10 release by Hfcm, starting from 4.1 × 10^−6^, with an IC_50_ = 4.6 × 10^−6^ M, similar to sildenafil, although one log higher. Data are derived from six separate experiments using distinct cell preparations. CXCL10 secretion is expressed as percent of IFNγ + TNFα-induced secretion (mean ± SE). *P < 0.05; **P < 0.01; ***P < 0.001.

Since sildenafil and MPA exerted a similar inhibitory effect on CXCL10 release in Hfcm, we compared drug potency *in vitro* in these cells. As we performed dose–response curves in Hfcm induced by inflammatory cytokines and incubated with scalar concentration of sildenafil or MPA, for comparison, we found that sildenafil decreased, in a dose-dependent manner, cytokine-induced CXCL10 secretion, significantly from 5 × 10^−7^ M (*P* < 0.05, *P* < 0.01 or *P* < 0.001 vs. IFNγ + TNFα-induced secretion; Fig. 2a). MPA has been confirmed to inhibit CXCL10 secretion dose-dependently (Fig. 2b, *P* < 0.01, *P* < 0.001 vs. IFNγ + TNFα-induced secretion), as previously shown [34]. The calculated IC_50_ was 2.6 × 10^−7^ M for sildenafil and 4.6 × 10^−6^ M for MPA, in line with our previous results [34].

### Effect of Sildenafil onto CXCL10 Protein Secretion in Comparison with Other PDE Subtype Blockage in Hfcm

**Fig. 3:**
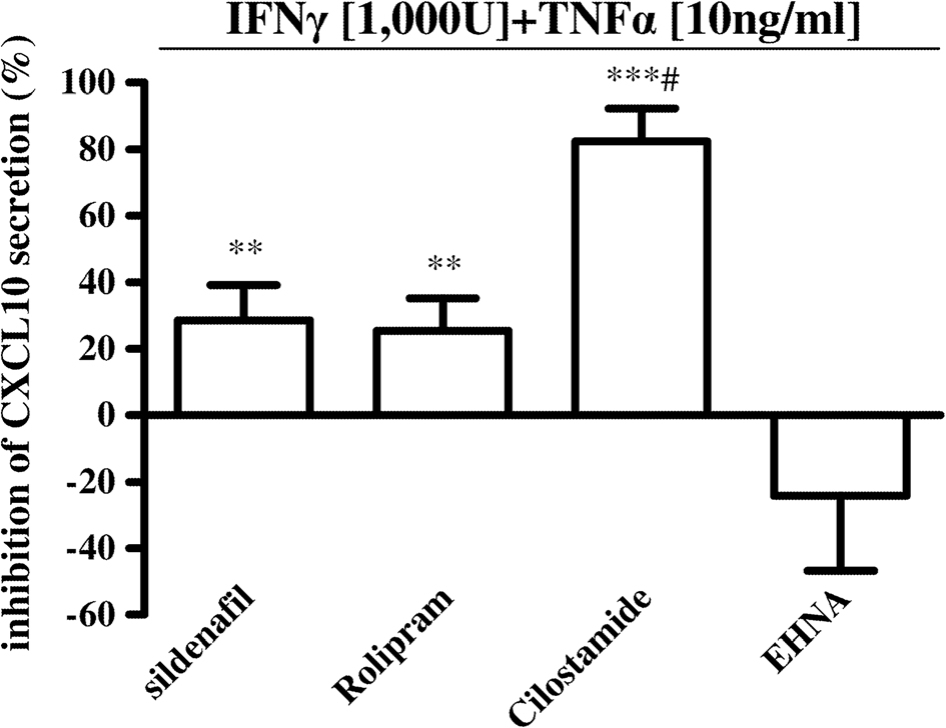
Effect of different PDE subtype blockage on cytokine-induced CXCL10 release by human cardiomyocytes. The specific blockage for 24 h of PDE3 activity with cilostamide (1 μM) almost blunted IFNγ + TNFα-induced CXCL10 release by human cardiomyocytes (82.4 ± 9.9 % inhibition, ***P < 0.001, ^#^ *P* < 0.05 vs. sildenafil treatment); PDE5 and PDE4 inhibition by sildenafil (1 μM) and rolipram (10 μM), respectively, significantly inhibited CXCL10 secretion (28.6 ± 10.7 and 25.5 ± 9.8 % inhibition, respectively, **P < 0.01); PDE2 blockage with EHNA (10 μM) had no significant effect (−24.0 ± 22.6 % inhibition). Results (mean ± SE) are expressed as inhibition of CXCL10 secretion, percent of IFNγ + TNFα-induced release, taken as 100 %. Data are obtained from experiments with four different cell preparations.

As we found that human cardiomyocytes were significantly targeted by sildenafil in terms of CXCL10 inhibition at protein and gene level, we compared cytokine-induced CXCL10 release by Hfcm after PDE subtype 5, 4, 3, and 2 specific blockage (Fig. 3). CXCL10 significantly decreased after PDE5 and PDE4 inhibition by sildenafil and rolipram, respectively (*P* < 0.01). PDE3 inhibition with cilostamide almost abrogated IFNγ + TNFα-induced CXCL10 secretion (*P* < 0.001), while PDE2 blockage with EHNA exerted no effect.

### Effect of Sildenafil on Hfcm, Hfaec, and PBMC Viability

**Fig. 4:**
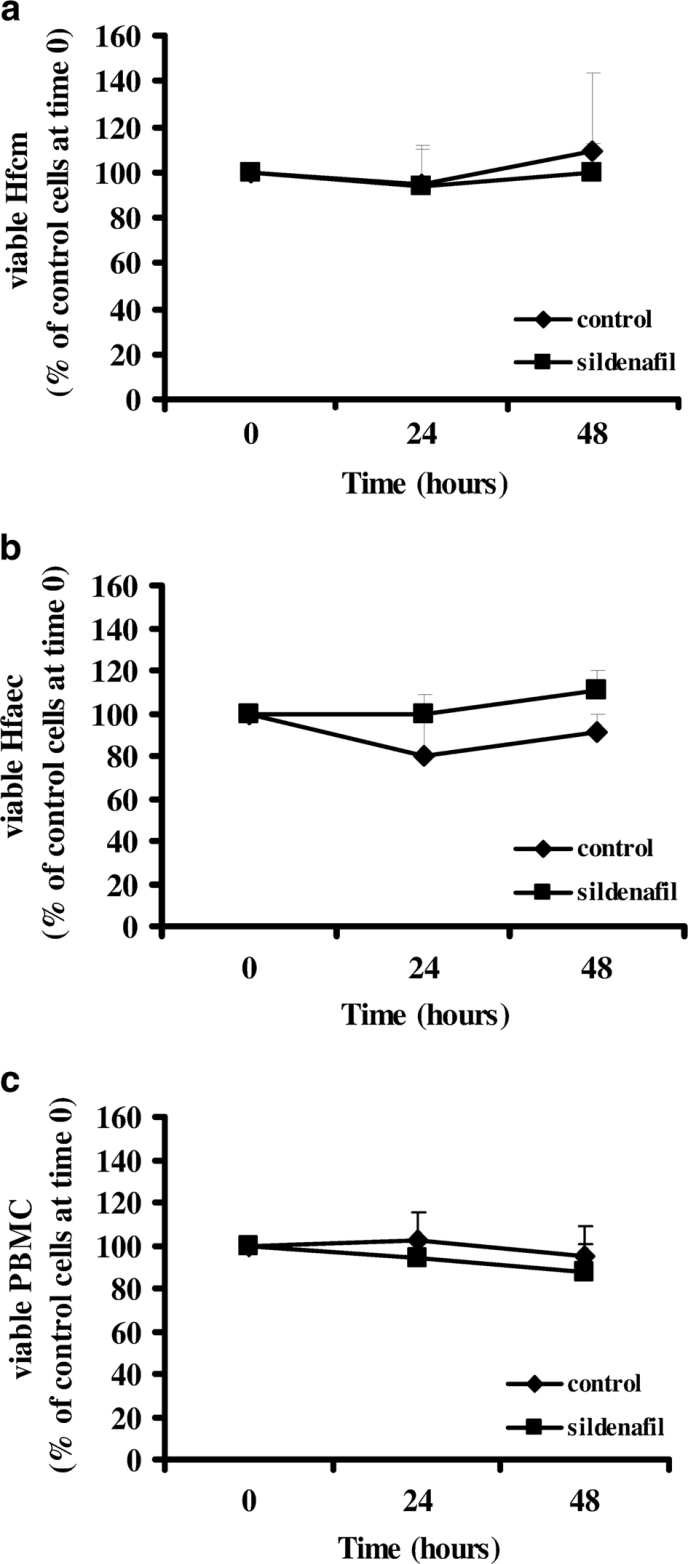
Effect of sildenafil on human cardiac, endothelial and immune cell viability. **a** Hfcm cell viability did not change after 24 or 48 h treatment with sildenafil (1 μM, *square*) vs. control cells (*rhombus*). **b** Hfaec viability was unvaried after 24- or 48-h treatment with sildenafil (1 μM, *square*) vs. control cells (*rhombus*). **c**. PBMC viability after 24–48 h did not change with sildenafil (1 μM, *square*) vs. control cells (*rhombus*). Results derived from at least four different cell preparation for each cell type and are expressed as percent of control cells (taken as 100 %) at time 0.

Sildenafil did not affect Hfcm, Hfaec and PBMC viability after 24–48 h as compared with control cells, Fig. 4a, b, c.

### Effect of Sildenafil onto CXCL10 Serum Level in Diabetic Cardiomyopathy

**Fig. 5:**
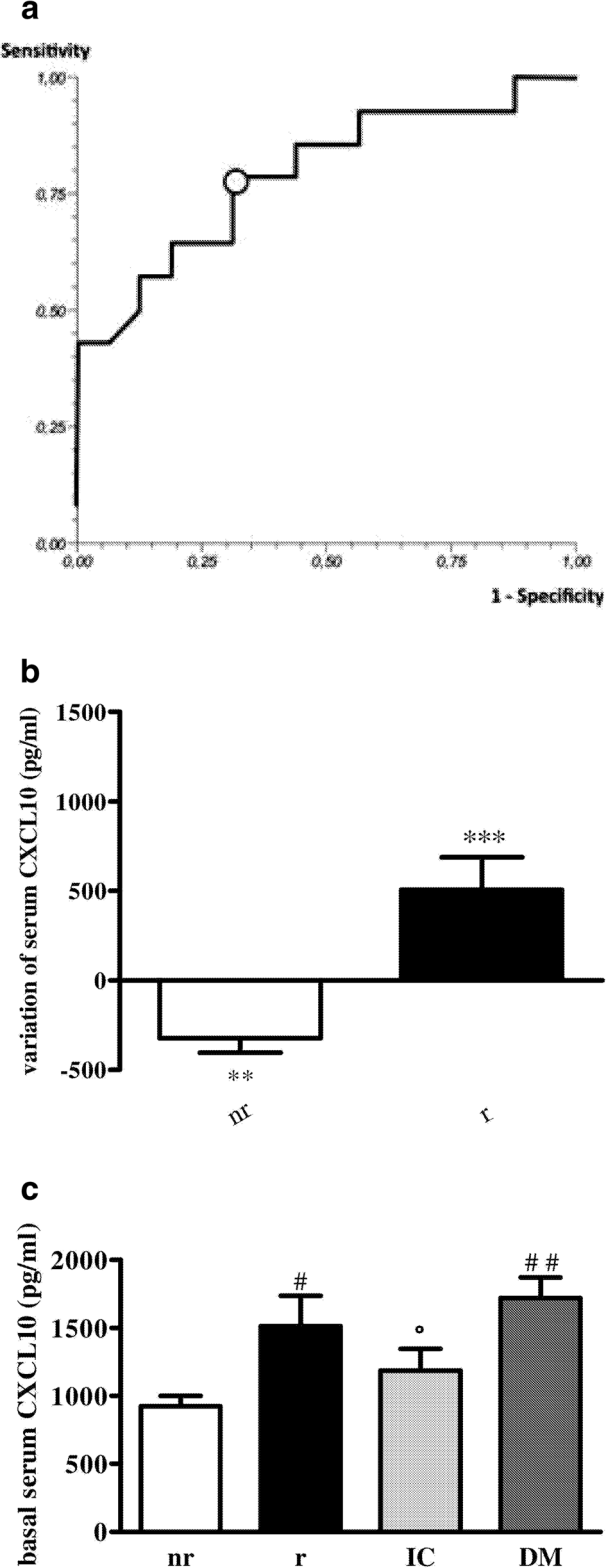
Effect of sildenafil on circulating CXCL10 level in diabetic cardiomyopathy. **a** Receiver operating characteristic (ROC) analysis evidenced that serum CXCL10 = 930 pg/ml (cut-off value, *open circle*) had good capability to discriminate subjects who did or did not respond to sildenafil administration, in terms of circulating chemokine decrease. The area under the ROC curve (AUC) resulted 0.80 (95 % confidence interval = 0.584–0.996, *P* < 0.01) with a sensitivity of 0.78 and a specificity of 0.69. **b** Subjects have been categorized as drug responders (r) and non-responders (nr) to sildenafil treatment. Positive or negative variations in circulating CXCL10 have been observed after sildenafil intake vs. basal level (504.84 ± 182.6 pg/ml, −323.93 ± 82 pg/ml). Data are expressed as serum CXCL10 change, in picogram per milliliter vs. basal level (mean ± SE); (**P < 0.01, ***P < 0.001, vs. basal level). **c** r and nr subgroups showed significant difference in CXCL10 basal level (1514.92 ± 221.22 pg/ml vs. 923.45 ± 76.71 pg/ml, respectively ^##^ *P* < 0.01); CXCL10 level in diabetes (DM, 1719.46 ± 151.4 pg/ml) was similar to basal level in r subgroup and higher than basal level in nr subgroup (^##^ *P* < 0.01). Subjects with non-diabetic cardiomyopathy (IC) displayed circulating CXCL10 (1186.39 ± 162.38 pg/ml) lower than DM and r (°*P* < 0.05). Data are expressed as basal serum CXCL10 level, in picogram per milliliter (mean ± SE).

To verify whether PDE5 inhibition might affect *in vivo* Th1-associated conditions, we measured CXCL10 level in blood of subjects with diabetic cardiomyopathy before and after sildenafil intake. Receiver operating characteristic (ROC) analysis (Fig. 5a) showed that circulating CXCL10 = 930 pg/ml is the cut-off value retaining a good ability to discriminate between patients with positive or null/negative response to sildenafil (Fig. 5b): this means that drug intake induced a significant decrease of chemokine level only in subjects with circulating CXCL10 over 930 pg/ml (positive change, *P* < 0.001); subjects with CXCL10 below cut-off value showed no change or an increase (null/negative change, *P* < 0.01) in serum chemokine level; subjects were then defined as (r) responders and non-responders (nr) to sildenafil.

CXCL10 level was significantly different in r and nr subgroups (*P* < 0.01, Fig. 5c);in particular, r subgroup showed CXCL10 serum level similar to subjects affected by diabetes without sings of cardiac disease (DM), but significantly different from subjects with non-diabetic cardiomyopathy (IC, *P* < 0.05 vs. DM and r; Fig. 5c).

## DISCUSSION

The main finding of this study is that sildenafil in human cardiomyocytes inhibited protein and gene expression of IFNγ + TNFα-induced chemokine CXCL10 (IC_50_ 2.6 × 10^−7^ M), which is a critical trigger of early Th1 immune/inflammatory response during cardiac diseases [12–17]. Sildenafil also significantly reduced inflammatory cytokine-induced IL-6 secretion by Hfcm. Furthermore, sildenafil decreased CXCL10 blood level higher than 930 pg/ml (cut-off value) in diabetic subjects at cardiomyopathy onset.

Cardiovascular homeostasis is tightly linked to the immune/inflammatory status. Th1-type inflammatory dominance is considered the common link in several co-morbid conditions, such as diabetes, atherosclerosis, and heart dysfunction [1, 2]. A number of Th1-related inflammatory molecules, produced by different tissues and cells, have been shown to widely participate to disease initiation and progression, as well [12–17]. In this scenario, heart-related studies began to focus on the complex communications between cardiomyocytes and other cells—*i.e*., endothelial and inflammatory cells recruited to and residing within the heart. Th1-type cytokines and chemokines have been shown to be highly active mediators in cell-to-cell interactions through endocrine, paracrine or autocrine pathways, triggering immune/inflammatory response [6, 9, 37] at disease onset. In particular, previous human *in vivo* and *in vitro* studies reported that CXCL10, released by cardiac, endothelial and immune cells challenged by inflammatory conditions could be eligible as reliable marker and therapeutic target at early stages of heart disease, when Th1-type dominance prevails [12, 13, 15, 20, 38].

Recently, the use of oral PDE5i to treat different cardiac diseases has been sustained and encouraged by several animal and clinical evidences [23, 31, 37, 39–44]. In particular, sildenafil has been evolving from an anti-angina drug to a treatment for ED and, afterward, it has been approved for primary pulmonary hypertension [38, 45–47]. PDE5i beneficial effects seem undoubtedly linked to sildenafil-induced vascular tone regulation: through PDE5 selective inhibition, the release of endogenous cardioprotective molecules (*i.e*., adenosine and bradykinin) from vascular bed would be promoted, cause/effect relationships in heart tissue accomplished and cardiac cell protection indirectly achieved [48].

In a rat model of pulmonary hypertension, novel mechanisms for sildenafil protection have been shown to relay on preservation of lung morphology, attenuation of inflammatory paths with reduction of several cytokine release, finally ending in a decrease of inflammatory cell infiltration [49].

We provide the first evidence that sildenafil in human cardiomyocytes could counteract CXCL10 protein release with a potency similar to MPA—although with an IC_50_ of one log lower—and chemokine-specific mRNA expression, as well.

We have previously reported that in Hfcm CXCL10 secretion is mediated by the nuclear transcription factor-kB (NF-kB) and that drug-induced reduction of CXCL10 release by human cardiomyocytes is associated to a block of NF-kB nuclear translocation [20, 38].

We could speculate that in Hfcm sildenafil is able to repress NF-kB activity acting at transcription level *via* cGMP stabilization, as shown in previous studies [50, 51]; in line with this hypothesis, cGMP level increased in Hfcm after sildenafil, similarly to Hfaec (not shown); our ongoing study aims to clarify those underlying biomolecular mechanism(s).

Conversely, in Hfaec stimulated by proinflammatory cytokines, sildenafil did not affect CXCL10 mRNA expression while chemokine protein release was reduced, albeit to significantly less extent than in Hfcm. In TNFα-stimulated human endothelial cells promoter targeting and transcriptional activation of CXCL10 has been shown to depend on NF-kB activation as well [52]. Changes in transcription not always reflect changes in protein expression [53] and differences in protein/mRNA expression may result from gene transcription, specific mRNA stability or translation [54–62]. A deregulation at post-trascriptional level, concerning, *i.e*., mRNA processing/maturation or stability could result in reduction of translated CXCL10 protein in Hfaec treated with sildenafil [63].

In PHA-activated PBMC sildenafil affected CXCL10 expression neither at protein nor at gene level. Similarly, sildenafil did not exert any significant effect onto CXCL10 released by activated monocytes and macrophages. The lack of significant effects onto immune cells would suggest the exclusion of possible specific modulating effects on immune system by sildenafil.

In this research, we have screened CXCL10 mRNA expression in Hfcm, Hfaec, and PBMC 24 h after stimulation, at time when the maximal induction of chemokine protein release occurs in all cell types [15]; however, the discrepancy found in CXCL10 protein/mRNA expression after sildenafil in cardiac, endothelial and immune cells cannot exclude some experimental conditions, since incubation/harvest time could be differentially optimal for mRNA detection depending on different cell types. Our future gene studies will include fine-tuned time-course assays.

Concerning *in vivo* data, we show that in diabetic cardiomyopathy serum CXCL10 positive or negative variation after sildenafil intake categorized subjects as drug responders vs. non-responders; in fact, as revealed by ROC analysis, CXCL10 serum level equal or higher than cut-off value (930 pg/ml) is significantly predictive of response to sildenafil. This means that subjects (half number) showing basal circulating CXCL10 level higher than the cut-off value responded to sildenafil treatment with a significant decrease of the chemokine in blood (positive variation); hence, those subjects have been defined as “responders”. No decrease in serum chemokine level (null/negative variation) after drug intake has been found in subjects with basal circulating CXCL10 levels lower than 930 pg/ml, and, therefore, named “non-responders”.

As we evaluated sex- and age-matched subjects affected by diabetes or cardiomyopathy at the same disease stage as patients with comorbidity, we found that cardiomyopathy *per se* does not display high systemic CXCL10 with sera values similar to non responders (and healthy subjects, not shown) at variance with diabetes. This latter result could be due to the wide involvement of this chemokine in diabetes-associated vascular bed alterations [37, 64], where CXCL10, among other Th1-type biomediators, is described to be increased (devaraj et al. 2009 cytokine) and responsible for triggering vascular alteration and damage [14, 64].

We could speculate that in some subjects, regardless DM or DM-induced cardiomyopathy, the higher CXCL10 level in blood likely derives from different cell and tissue types and could mirror a wider activation and amplification of the Th1-type response, during which sildenafil seems to be effective while it is neutral when CXCL10 is below the cut-off level, as from our ROC analysis data.

Interestingly, a recent meta-analysis reports that sildenafil causes significant improvements only in subjects with heart failure (HF) and reduced ejection fraction [65] without any beneficial effect in HF subjects with preserved ejection fraction [66, 67]. So far, it is likely that PDE5 inhibition would be capable to oppose “destabilized” homeostatic conditions with an anti-inflammatory action potentially associated with clinical favorable outcome.

This topic is presently under debate along with the presence of PDE5 expression in heart [66].

A recent study by Degen et al. [68] was unable to detect PDE5 protein expression in tissues from healthy and failing heart of humans and addressed the lack of therapeutic effect of sildenafil in HF—shown by RELAX trial or similar studies [67, 69]—on the basis of PDE5 low level/absence. Nevertheless, PDE5 upregulation has been detected in inflammation-related processes, *i.e*., oxidative stress and pressure overload hypertrophy [70–74], and would explain the favorable effects on cardiac function achieved by PDE5 inhibition. So far, the current hypothesis is that PDE5 expression is undetectable in normal myocardium, upregulated during hypertrophy and downregulated in end-stage HF [68]. In this light, human heart tissue collection obtained at time of heart transplantation in end-stage HF, when PDE5 expression is downregulated below detection limits, would justify the absence of specific protein signal as shown by Degen et al. [68].

In cell lysates from Hfcm, we have detected PDE5-positive-specific signal, similarly to Hfaec and all immune cell tested by western blot analysis performed with the same antibody reported by Degen et al. [68]. It is possible that specific signal detection could be technically easier using cell lysates from virtually pure culture of human cardiomyocytes, which require sample processing different from tissue lysates.

## CONCLUSIONS

The potential advantage of PDE5i use in cardiovascular diseases has been assumed to be based on counteracting some pathophysiologic perturbations, *i.e*., attenuation of adrenergic stimulation, reduction of ventricular–vascular stiffening and maladaptive chamber remodeling, together with improvement of endothelial function, hemodynamics and enhancement of renal responsiveness [75, 76].

In line with previous data showing that sildenafil is associated with cardioprotection and reduction of multiple circulating inflammatory cytokine levels in Th1-driven conditions [31, 49], herein, we reported that sildenafil reduced high circulating CXCL10, likely deriving from multiple cell types activated during Th1-driven inflammation.

Our results obtained in human cardiomyocytes and endothelial cells suggest that sildenafil can counteract Th1-driven processes by targeting CXCL10 at local cellular level.

This observation seems particularly relevant since CXCL10, engaged in cardiac disease onset, is not linked to generic inflammatory status but critically initiates the response to Th1-driven inflammatory challenge and triggers a self-detrimental loop [12–16]. Remarkably, the specific inhibition of PDE3, that is well recognized to retain cardioprotective effects [29], almost blunted CXCL10 secretion by Hfcm as well. However, it is unlikely that sildenafil effect may be limited to CXCL10; indeed, we might hypothesize that the inhibition of IL-6 release by human cardiac cells might contribute as well to sildenafil-induced cardioprotective effects. IL-6, as a cardiomyocyte-derived cytokine, has been shown to start positive feedback circuits and promote cytokine amplification, thereby contributing to chronic inflammatory state establishment [77]. Conversely, our results in human cardiomyocytes on modulation of IL-8 did not evidence significant effects after sildenafil. However, further fine-tuned investigations on possible sildenafil effects onto those and other tissue/systemic cytokines are necessary, also considering that temporal regulation of those biomediators might result in protective or pathogenic effects, as recently reported for IL-6 [78].

Finally, our *in vitro* and *in vivo* study present some limitations, *i.e*., the limited number of biomediators analyzed, or the lack of group of subjects with diabetes mellitus or cardiomyopathy alone treated with sildenafil. In conclusion, albeit we cannot correlate the results obtained in cells and in patients, our data showed that sildenafil targets CXCL10 in Th1-type-dominant conditions and contribute to confirm the anti-inflammatory properties of this drug.

Further *in vivo* investigations, including a larger number of cytokine types and patients, different stages of cardiomyopathy, longer treatment with sildenafil and follow-up evaluation are mandatory as well as additional *in vitro* studies on biomolecular mechanism(s) in order to elucidate intracellular target(s) in cell target-based PDE5i pharmacokinetic studies. In light of those preliminary evidences, we speculate that sildenafil could be a new therapeutic option to control cardiomyopathy onset/progression in diabetic subjects while CXCL10 could represent a novel tool to identify patients to treat since early stages.

## ELECTRONIC SUPPLEMENTARY MATERIAL

Below is the link to the electronic supplementary material.

Fig. S1 Effect of sildenafil on cytokine-induced IL-6 and IL-8 secretion in human cardiac cells in comparison with different immunosuppressors. **a** In Hfcm IFNγ + TNFα-induced IL-6 protein secretion was significantly decreased after 24-h incubation with sildenafil or MeP (38.4 ± 7.4 and 72.4 ± 7.7 % inhibition, ***P < 0.001); CsA and MPA did not exert any effect. **b** Twenty-four-hour incubation with sildenafil or MPA did not change IFNγ ± TNFα-induced IL-8 protein secretion in Hfcm; MeP, and CsA significantly decreased this chemokine (52.6 ± 9.7 and 29.8 ± 18.2 % inhibition; ***P < 0.001, *P < 0.05, respectively). Results (mean ± SE) are expressed as inhibition of IL-6 and IL-8 secretion percent of IFNγ ± TNFα-induced release, taken as 100 %. Data are obtained from five to nine experiments using different cell preparations. (GIF 58 kb)

High Resolution Image (TIFF 78 kb)

Fig. S2 Effect of sildenafil on LPS-induced CXCL10 secretion in human monocytes/macrophages lineage in comparison with different immunosuppressors. **a** Sildenafil, CsA, and MPA did not significantly affect CXCL10 release by LPS-activated human monocytes after 48 h, at variance with MeP (48.8 ± 6.3 % inhibition; *P < 0.05). **b** In human macrophages, sildenafil and all tested drugs did not exert any significant effect on LPS-induced CXCL10 protein secretion. **c** Flow cytometric analysis of CD14^+^ cells in PBMC before and after positive selection. Microbeads conjugated to anti-CD14 monoclonal antibodies were used for the positive selection of human monocytes by PBMC from healthy donors. **a** Expression of CD14 in PBMC before positive selection. **b** Expression of CD14 after positive selections. *Numbers* within the hystograms indicate the percentage (more than 90 %) of CD14^+^ cells. Results (mean ± SE), in **a** and **b**, are expressed as inhibition of CXCL10 secretion percent of LPS-induced release, taken as 100 %. Results in **c** show the number of the events (*Y*-axis) and antiCD14-FITC conjugated (FL-1) signal in log scale (*X*-axis). Data are obtained from four separate experiments. (GIF 43 kb)

High Resolution Image (TIFF 101763 kb)

## Abbreviations

BMI: Body mass index
HOMA: Homeostatic model assessment
HbA1c: Glycated hemoglobin
BP: Blood pressure
LVMi: Left ventricular mass index
EDVi: End-diastolic volume index
