# Supplementary figures and images for: Phosphodiesterase Type 5 Inhibitor Sildenafil Decreases the Proinflammatory Chemokine CXCL10 in Human Cardiomyocytes and in Subjects with Diabetic Cardiomyopathy
Source: Inflammation. 2016 May 10;39:1238–52. doi: 10.1007/s10753-016-0359-6 (PMC4883282; doi:10.1007/s10753-016-0359-6)

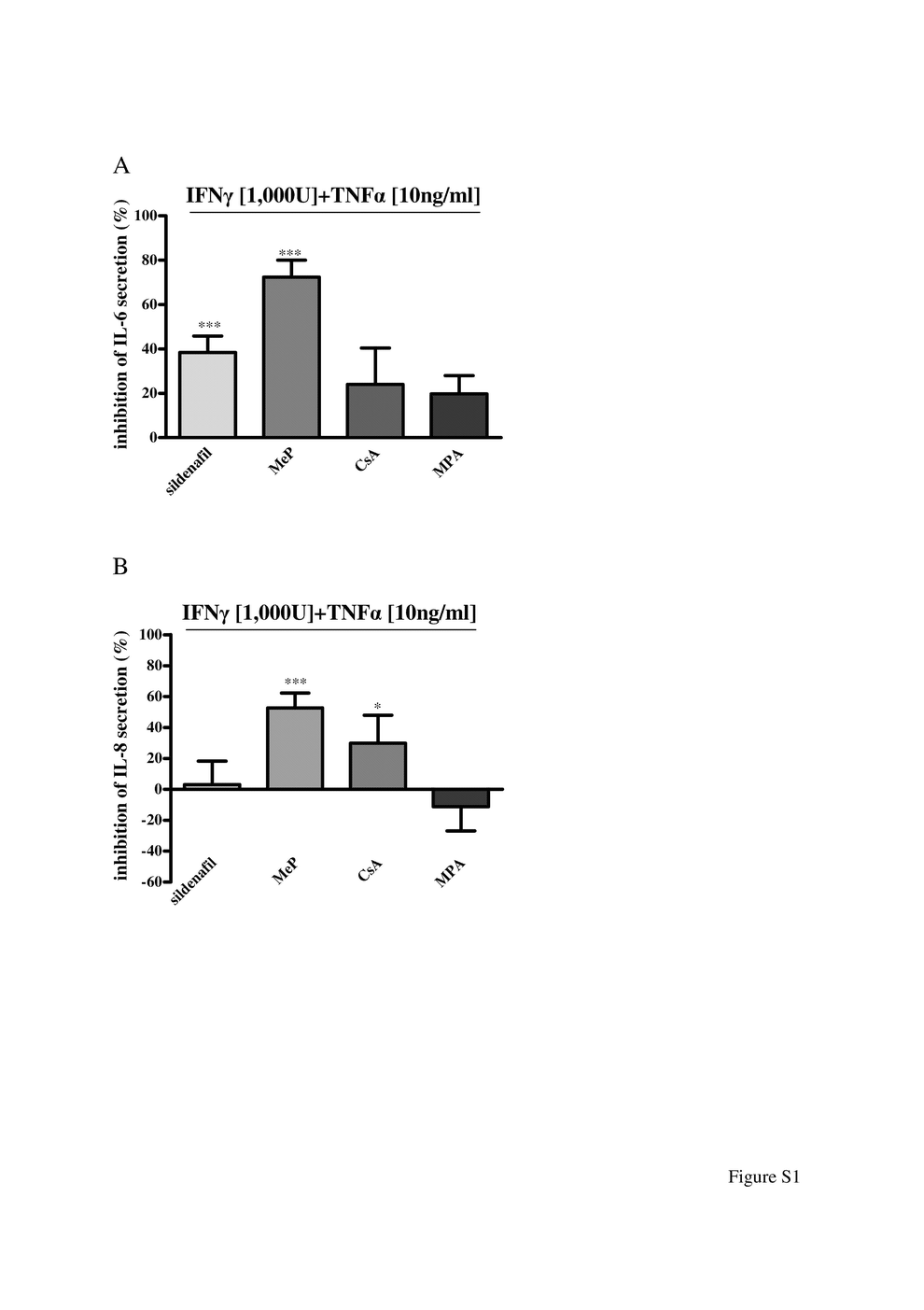

Supplement: Supplementary file 1 — Effect of sildenafil on cytokine-induced IL-6 and IL-8 secretion in human cardiac cells in comparison with different immunosuppressors. a In Hfcm IFNγ + TNFα-induced IL-6 protein secretion was significantly decreased after 24-h incubation with sildenafil or MeP (38.4 ± 7.4 and 72.4 ± 7.7 % inhibition, ***P < 0.001); CsA and MPA did not exert any effect. b Twenty-four-hour incubation with sildenafil or MPA did not change IFNγ ± TNFα-induced IL-8 protein secretion in Hfcm; MeP, and CsA significantly decreased this chemokine (52.6 ± 9.7 and 29.8 ± 18.2 % inhibition; ***P < 0.001, *P < 0.05, respectively). Results (mean ± SE) are expressed as inhibition of IL-6 and IL-8 secretion percent of IFNγ ± TNFα-induced release, taken as 100 %. Data are obtained from five to nine experiments using different cell preparations. (GIF 58 kb) [file 10753_2016_359_Fig6_ESM.gif]

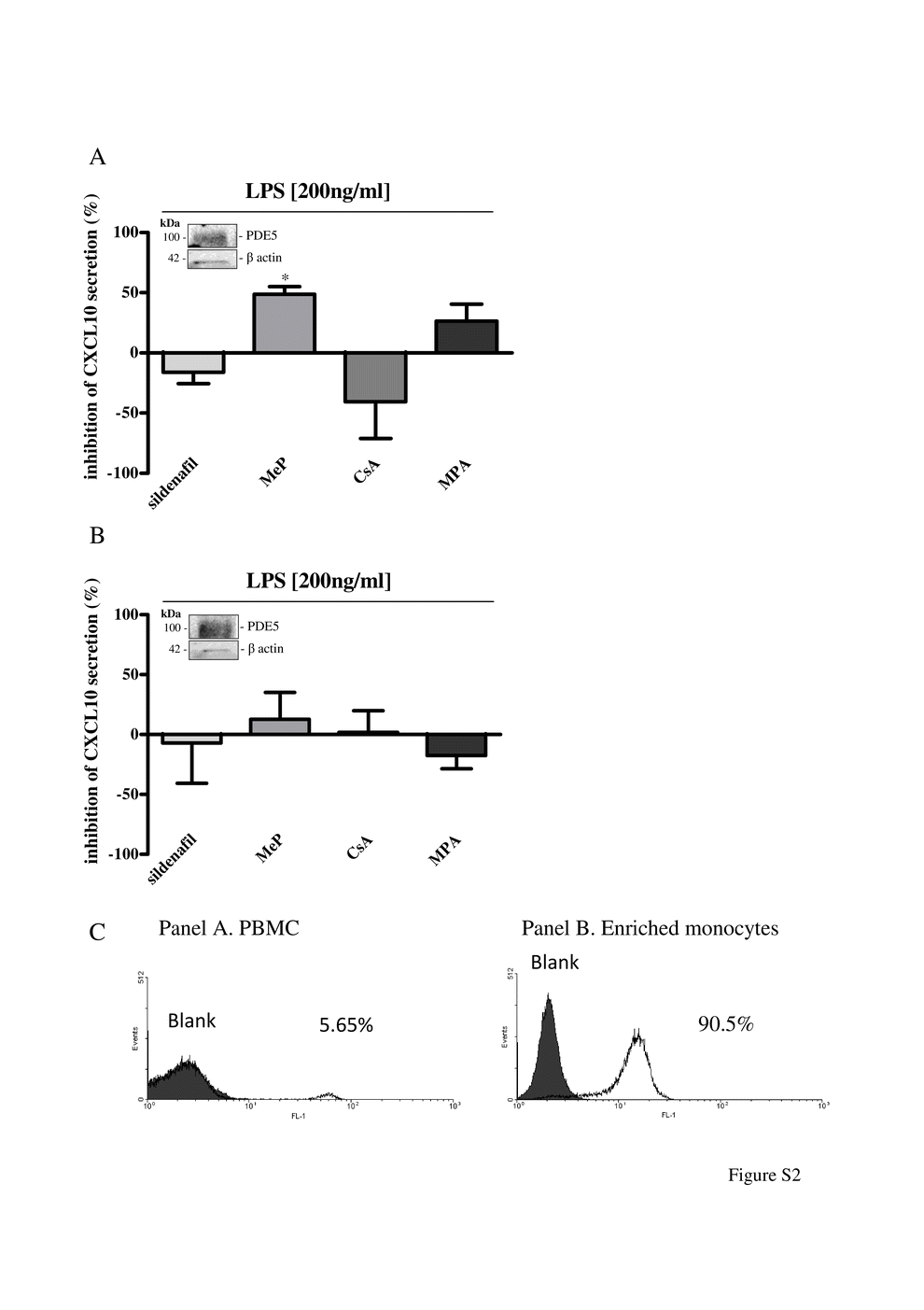

Supplement: Supplementary file 3 — Effect of sildenafil on LPS-induced CXCL10 secretion in human monocytes/macrophages lineage in comparison with different immunosuppressors. a Sildenafil, CsA, and MPA did not significantly affect CXCL10 release by LPS-activated human monocytes after 48 h, at variance with MeP (48.8 ± 6.3 % inhibition; *P < 0.05). b In human macrophages, sildenafil and all tested drugs did not exert any significant effect on LPS-induced CXCL10 protein secretion. c Flow cytometric analysis of CD14+ cells in PBMC before and after positive selection. Microbeads conjugated to anti-CD14 monoclonal antibodies were used for the positive selection of human monocytes by PBMC from healthy donors. a Expression of CD14 in PBMC before positive selection. b Expression of CD14 after positive selections. Numbers within the hystograms indicate the percentage (more than 90 %) of CD14+ cells. Results (mean ± SE), in a and b, are expressed as inhibition of CXCL10 secretion percent of LPS-induced release, taken as 100 %. Results in c show the number of the events (Y-axis) and antiCD14-FITC conjugated (FL-1) signal in log scale (X-axis). Data are obtained from four separate experiments. (GIF 43 kb) [file 10753_2016_359_Fig7_ESM.gif]
